# Exocrine pancreatic function in children with Alagille syndrome

**DOI:** 10.1038/srep35229

**Published:** 2016-10-17

**Authors:** Dorota Gliwicz, Irena Jankowska, Aldona Wierzbicka, Anna Miśkiewicz-Chotnicka, Aleksandra Lisowska, Jarosław Walkowiak

**Affiliations:** 1 The Children’s Memorial Health Institute, Department of Gastroenterology, Hepatology, Feeding Disorders and Pediatrics, Warsaw, Poland; 2 The Children’s Memorial Health Institute, Department of Biochemistry and Experimental Medicine, Warsaw, Poland; 3 Poznan University of Medical Sciences, Department of Pediatric Gastroenterology and Metabolic Diseases, Poznań, Poland

## Abstract

Alagille syndrome (AGS) is often associated with symptoms of maldigestion, such as steatorrhea, hypotrophy and growth retardation. Exocrine pancreatic insufficiency was proposed as the underlying cause. We aimed to assess the exocrine pancreatic function with the use of different methods in AGS patients. Concentrations of fecal elastase-1 (FE1) and fecal lipase (FL) activities were measured in 33 children with AGS. The C-mixed triglyceride breath test (MTBT) in a subgroup comprising 15 patients. In all patients studied, FE1 concentrations and FL activities were normal. Abnormal MTBT results were documented in 4 (26.7%) patients. The FE1 and FL levels in MTBT-positive and MTBT-negative children did not differ. The results of this research do not confirm the presence of exocrine pancreatic dysfunction in AGS patients. Routine screening for exocrine pancreatic insufficiency of this group of patients is not necessary.

Alagille syndrome (AGS) is a rare multiorgan disease inherited in an autosomal dominant pattern, caused by defects in the Notch signaling pathway. It is one of the most common, inherited liver diseases. The majority of cases are due to mutations in the *JAGGGED1 (JAG1*) gene, encoding a ligand for Notch receptors. A small percentage of cases result from mutations in *NOTCH2*^1^. The disease has five major features: chronic cholestasis secondary to the paucity of intrahepatic bile ducts, cardiovascular abnormalities mainly affecting the pulmonary outflow tract, skeletal defects (most frequently butterfly vertebrae), ophthalmologic anomalies (most often posterior embryotoxon) and characteristic facial features. These are often accompanied by other minor symptoms, such as renal and intracranial vascular problems, hypercholesterolemia and growth retardation.

Exocrine pancreatic insufficiency is also described in the literature as a pathology found more often in AGS patients than in the general population^2^,^3^,^4^. Indeed, some symptoms observed frequently in children with AGS, such as growth retardation, hypotrophia or steatorrhea may be ascribed to the insufficient enzymatic function of the pancreas. The presence of pathology in the pancreas also seems likely in view of the well-established expression of the *JAG1/Notch* signaling pathway genes in this organ^5^.

Despite these findings, cited in consecutive papers, no guidelines concerning the necessity to screen for exocrine pancreatic insufficiency or enzyme supplementation in AGS patients have been developed. Recently, Kamath *et al.*^6^ documented that the fecal elastase-1 (FE1) concentration was normal in the vast majority of 42 subjects with AGS^6^. However, the authors did not exclude isolated lipase deficiency. Therefore, in this research we evaluated pancreatic exocrine function in AGS subjects and we measured their lipase output. In a subset of patients, we also determined the biological effects of exocrine pancreatic secretion using the stable isotope breath test – mixed triglyceride breath test.

## Results

**Figure 1:**
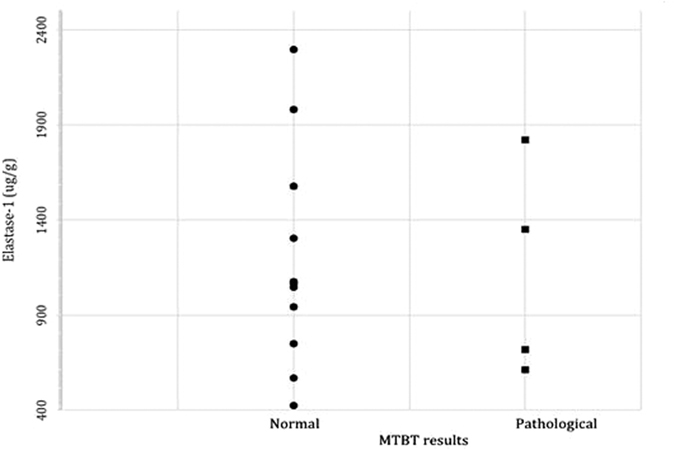
Distribution of fecal elastase-1 concentrations in patients with normal and pathological MTBT results. MTBT- C-mixed triglyceride breath test.

All study subjects were shown to have normal pancreatic secretion based on the FE1 test. FL activities were no different than those in the comparative group (158 < 81–395 > U/L)^7^. MTBT results were normal in 11 subjects and abnormal in the remaining 4 patients studied (26.7%) (Table 1). FE1 concentrations and FL activities did not differ between subgroups of subjects with normal and abnormal MTBT results (Fig. 1).

## Discussion

Pancreatic exocrine insufficiency has been suggested as one of the most common clinical features of AGS. Chong *et al.* carried out a secretin-pancreozymin stimulation test in 13 children with a paucity of interlobular bile ducts (11 with AGS) and found pancreatic insufficiency in 6 of them^2^. Emerick *et al.*, summarizing the clinical manifestation of 92 patients with AGS, documented a 42% incidence of abnormal fecal fat tests^3^. Subsequently, Rovner *et al.* – analyzing the causes of body hight and weight deficit in children with AGS – revealed an insufficient caloric intake, but also a very high incidence of steatorrhea, disclosed in 96% of the examined children^8^.

In 2009, Golson reported on several abnormalities within the pancreas, which appeared in mice examined after the experimental removal of both *JAGGED1* gene alleles. Among others, malformation of the pancreatic duct, fatty degeneration, fibrosis, pancreatic inflammation and insufficiency were described^4^. In a recent human study, Kamath *et al.* failed to document pancreatic exocrine insufficiency in 42 AGS patients using the FE1 test. However, the authors did not exclude isolated lipase deficiency^6^. Wen *et al.* assessed both FE1 concentrations and FL activities in stool and did not find any abnormalities. However, their study consisted of exclusively four subjects with AGS^9^. Therefore, in our research, we aimed to assess the exocrine pancreatic function of a larger subset of AGS subjects using the generally accepted indirect pancreatic test (FE1) as well as measuring lipase output. In addition, we performed MTBT to assess fat digestion and absorption.

The frequency of maldigestion as assessed using MTBT was documented to be moderate (26.7%) and definitely less frequent than in the previous studies^2^,^3^. However, none of the AGS subjects had abnormal FE1 results, suggesting exocrine pancreatic insufficiency as an underlying cause. What is really important, normal lipase secretion was documented – no different for those with or without maldigestion – suggesting that the noted steatorrhea is associated with other causative factors than exocrine pancreatic insufficiency. This research was carried out without direct control population, which could be considered as a potential limitation of this study. However, referring to available evidence, the FE1 results obtained in the present study could undoubtedly be referred to existing normal values^10^. Very good FE1 test sensitivity is limited to pancreatic insufficient patients and could be potentially limited in borderline fecal fat excretion. However, FE1 concentrations in subjects with normal and abnormal MTBT results were very similar. The lack of direct comparison between FE1/FL and fecal fat losses in all subjects studied could also be considered as a study limitation.

Lipase activities were additionally measured to exclude isolated lipase deficiency that could result in steatorrhea. Although the data on normal and abnormal FL activities are sparse, FL has not been validated as a diagnostic test for isolated lipase deficiency, and the sample-to-sample and day-to-day variation for FL is higher than for FE1, it seems to represent exocrine pancreatic secretion in terms of lipolytic function. The distribution of FL in AGS patients is very similar to that observed in a healthy population from our previous study and importantly, no sex or age-related relationships had been detected previously^7^. Therefore, bearing in mind some limitations, we can assume that the aim of the lipase assessment has been achieved. AGS growth retardation is not related to lipase deficiency as documented in the present study. However, it could be a part of whole syndrome and therefore, we aimed to exclude it.

How can the former reports, then, be justified? The secretin-pancreozymin stimulation test is a basic direct test in the diagnostic process of pancreatic insufficiency. It is based on the analysis of bicarbonate and enzyme concentrations in the pancreatic juice after secretin and pancreozymin stimulation. The pancreatic juice is obtained by duodenal aspiration. Although still the “golden standard” in pancreatology, it has some limitations. It is expensive and very invasive, so it cannot be routinely used for children. Additionally, it may not be reliable in patients with cholestasis, which is even more important for the context of our study^2^. A decreased concentration of bile acids in the intestinal content may cause lipase deactivation and, subsequently, false positive results of the stimulation test (suggesting a too low concentration of lipase in the pancreatic juice).

Lipid malabsorption is often observed in cholestatic children (PFIC-1 and PFIC-2)^11^,^12^. It is mainly due to a decreased secretion of bile acids to the digestive tract, which leads to steatorrhea, a deficiency of essential fatty acids and fat soluble vitamins. The overall clinical picture – diarrhea, failure to thrive – may suggest exocrine pancreatic insufficiency. Therefore, the fecal fat test will not properly verify a tentative diagnosis of pancreatic insufficiency in this group of patients^10^. Growth impairment has been described in about 50–90% of AGS patients (depending on the author)^3^,^8^,^13^. Its etiology is probably multifactorial. Insufficient caloric intake and malnutrition present important problems. However, the etiology must also be extracholestatic, as growth retardation in other cholestatic syndromes of children is not so significant. Other possible causes include genetic predisposition, bone anomalies and accompanying heart and kidney abnormalities. Apparently, pancreatic dysfunction is not one of the causative factors. Moreover, neither the tests assessing fecal fat excretion nor the MTBT should be used in assessing the exocrine pancreatic function in patients suffering from severe cholestatic liver disease.

To summarize, the results of this research do not confirm the presence of exocrine pancreatic dysfunction in AGS patients and therefore, routine screening for pancreatic insufficiency of this group of patients is not necessary.

## Material and Methods

The group of 33 patients with AGS and liver involvement, aged 1 to 21 years (median: 9; 1st–3rd quartile: 7–16), treated in the Department of Gastroenterology, Hepatology, Feeding Disorders and Pediatrics of the Children’s Memorial Health Institute in Warsaw was investigated between July 2010 and June 2011. All patients fulfilled the AGS diagnostic criteria. These criteria were: the presence of at least 3 out of the 5 major features (cholestatic liver disease obligatory); in patients with no identified *JAG1* mutation – additionally, the occurrence of ductopenia in the liver specimen or 4 out of 5 major features. A *JAG1* gene mutation was detected in 24 children. Six patients who had undergone liver transplantation were included in this research. The median bilirubin concentration at the time of the study was 0.8 mg/dl (1st–3rd quartile: 0.8–1.8). A table with clinical and molecular data of the examined patients has been placed in the supplementary information.

For standardization purposes, body mass index (BMI) and height were compared with Polish growth charts^14^. The median (quartile) height *z-score* was −1,55 (−2,37; −0,72) and median BMI *z score* was −0,93 (−1,45; −0,55).

FE1 concentrations and fecal lipase (FL) activities were measured for all 33 patients. The assessment of FE1 and FL levels were performed with the spectrophotometric method using commercially available ELISA tests (Schebo Biotech GmbH, Giessen, Germany and Alfa Diagnostics, San Antonio, TX, USA, respectively). FE1 values lower than 200 μg/g were classified as abnormal^15^,^16^,^10^. Since no normal FL values exist, the obtained results were compared to those obtained previously in a group of healthy subjects^7^.

15 patients from the study group additionally took a mixed-triglycerides breath test as described previously^17^. The results were expressed as a cumulative percentage dose recovery (CPDR). Values lower than 13% were considered to be abnormal. In most cases, the breath test was performed on the same day as the samples were collected. In the remaining subjects, the length of the period between the breath test and sample collection did not exceed one month.

Medians and interquartile ranges were determined for all values. The statistical significance of differences in the results of the pancreatic tests of patients with normal and abnormal MTBT was determined using the Whitney-Mann rank test. A similar analysis was performed to compare FL activities in the studied and comparative group^7^. A value of p < 0.05 was considered statistically significant. All statistical analyses were performed using Statistica 9.0 software.

The studies were carried out in accordance with the revised Declaration of Helsinki. Informed consent was obtained from the patients’ parents. The study was approved by the appropriate Bioethical Committees at Poznań University of Medical Sciences (decision 45/KBE/2009) and the Bioethical Committee of the Children’s Memorial Health Institute in Warsaw (decision 45/KBE/2009), Poland.

## Additional Information

**How to cite this article**: Gliwicz, D. *et al.* Exocrine pancreatic function in children with Alagille syndrome. *Sci. Rep.* **6**, 35229; doi: 10.1038/srep35229 (2016).

## Supplementary Material

Supplementary Information

## Figures and Tables

**Table 1:** Fecal enzyme and breath test results in the examined group.

	Mean ± SEM	Median	1st–3rd quartile
Fecal elastase-1 (μg/g)	1113 ± 80	1030	843–1304
Fecal lipase (U/L)	211 ± 37	188	42–332
CPDR in MTBT (%)	23.1 ± 13	21.7	14.0–33.3

CPDR – cumulative percentage dose recovery. MTBT- C-mixed triglyceride breath test.

## References

[b1] Turnpenny P. D. & Ellard S. Alagille syndrome: pathogenesis, diagnosis and management. Eur. J. Hum. Genet. EJHG 20, 251–257 (2012). 21934706 10.1038/ejhg.2011.181 PMC3283172

[b2] Chong S. K., Lindridge J., Moniz C. & Mowat A. P. Exocrine pancreatic insufficiency in syndromic paucity of interlobular bile ducts. J. Pediatr. Gastroenterol. Nutr. 9, 445–449 (1989). 2621522 10.1097/00005176-198911000-00009

[b3] Emerick K. M. *et al.* Features of Alagille syndrome in 92 patients: frequency and relation to prognosis. Hepatol. Baltim. Md 29, 822–829 (1999). 10.1002/hep.510290331 10051485

[b4] Golson M. L., Loomes K. M., Oakey R. & Kaestner K. H. Ductal malformation and pancreatitis in mice caused by conditional Jag1 deletion. Gastroenterology 136, 1761–1771.e1 (2009). 10.1053/j.gastro.2009.01.040 19208348

[b5] Crosnier C. *et al.* JAGGED1 gene expression during human embryogenesis elucidates the wide phenotypic spectrum of Alagille syndrome. Hepatol. Baltim. Md 32, 574–581 (2000). 10.1053/jhep.2000.16600 10960452

[b6] Kamath B. M., Piccoli D. A., Magee J. C. & Sokol R. J. Pancreatic insufficiency is not a prevalent problem in Alagille syndrome. J. Pediatr. Gastroenterol. Nutr. 55, 612–614 (2012). 22614108 10.1097/MPG.0b013e31825eff61 PMC3666161

[b7] Walkowiak J. *et al.* Faecal elastase-1 test is superior to faecal lipase test in the assessment of exocrine pancreatic function in cystic fibrosis. Acta Paediatr. Oslo Nor.1992 93, 1042–1045 (2004). 10.1111/j.1651-2227.2004.tb02715.x 15456193

[b8] Rovner A. J. *et al.* Rethinking growth failure in Alagille syndrome: the role of dietary intake and steatorrhea. J. Pediatr. Gastroenterol. Nutr. 35, 495–502 (2002). 12394373 10.1097/00005176-200210000-00007

[b9] Wen W.-H. *et al.* Fecal elastase 1, serum amylase and lipase levels in children with cholestasis. Pancreatol. Off. J. Int. Assoc. Pancreatol. IAP Al 5, 432–437 (2005). 10.1159/000086545 15985768

[b10] Walkowiak J. *et al.* Indirect pancreatic function tests in children. J. Pediatr. Gastroenterol. Nutr. 40, 107–114 (2005). 15699676 10.1097/00005176-200502000-00001

[b11] Walkowiak J. *et al.* Exocrine pancreatic function in children with progressive familial intrahepatic cholestasis type 2. J. Pediatr. Gastroenterol. Nutr. 42, 416–418 (2006). 16641580 10.1097/01.mpg.0000218154.26792.6a

[b12] Walkowiak J. *et al.* Normal levels of serum pancreatic enzymes in patients with progressive familial intrahepatic cholestasis type 2. Acta Biochim. Pol. 57, 573–575 (2010). 21140002

[b13] Quiros-Tejeira R. E. *et al.* Variable morbidity in alagille syndrome: a review of 43 cases. J. Pediatr. Gastroenterol. Nutr. 29, 431–437 (1999). 10512403 10.1097/00005176-199910000-00011

[b14] Palczewska I. & Niedźwiecka Z. [Somatic development indices in children and youth of Warsaw]. In Polish. Med. Wieku Rozwoj. 5, 18–118 (2001). 11675534

[b15] Walkowiak J. Faecal elastase-1: clinical value in the assessment of exocrine pancreatic function in children. Eur. J. Pediatr. 159, 869–870 (2000). 11079208 10.1007/s004310000536

[b16] Walkowiak J. *et al.* Fecal elastase-1 cut-off levels in the assessment of exocrine pancreatic function in cystic fibrosis. J. Cyst. Fibros. Off. J. Eur. Cyst. Fibros. Soc. 1, 260–264 (2002). 10.1016/s1569-1993(02)00096-6 15463824

[b17] Lisowska A. *et al.* Small intestine bacterial overgrowth does not correspond to intestinal inflammation in cystic fibrosis. Scand. J. Clin. Lab. Invest. 70, 322–326 (2010). 20560844 10.3109/00365513.2010.486869

