# Supplementary material for: Exocrine pancreatic function in children with Alagille syndrome
Source: Sci Rep. 2016 Oct 17;6:35229. doi: 10.1038/srep35229 (PMC5066201; doi:10.1038/srep35229)
Supplement: Supplementary Information [file srep35229-s1.pdf]

Exocrine pancreatic function in children with Alagille syndrome

Dorota Gliwicz-Miedzińska[1], Irena Jankowska[1], Aldona Wierzbicka[2], Anna Miśkiewicz-Chotnicka[3], Aleksandra Lisowska[3], Jarosław Walkowiak[*3]

[1]The Children's Memorial Health Institute, Department of Gastroenterology, Hepatology, Feeding Disorders and Pediatrics, Warsaw, Poland, [2]The Children's Memorial Health Institute, Department of Biochemistry and Experimental Medicine, Warsaw, Poland, [3]Poznan University of Medical Sciences, Department of Pediatric Gastroenterology and Metabolic Diseases, Poznań, Poland

Table. Clinical and molecular data of the examined patients

**Patient**	**cholestasis**	**ductopenia**	**cardiovascular abnormality**	**ophthalmic anomaly**	**butterfly vertebrae**	**facial features**	***JAG1* mutation**	**mutation type**	**height z-score**	**BMI z-score**
1	*yes*	yes	PPS	*yes*	no	yes	ND (after MLPA)		0,01	-0,06
**2**	yes	yes	no	no	*yes*	yes	yes	nonsense	-2,41	-1,45
**3**	yes	yes	PPS	*yes*	no	yes	yes	missense	-1,89	-0,93
**4**	yes	yes	PPS	*yes*	no	yes	ND (after MLPA)		-0,52	-1,98
**5**	yes	yes	no	*yes*	no	yes	yes	missense	-2,37	-0,74
**6**	yes	NE	PPS	*yes*	*yes*	yes	yes	missense	-1,55	-0,23
**7**	yes	NE	HP	*yes*	no	yes	yes	frameshift	-1,33	-1,32
**8**	yes	yes	PPS	*no*	no	yes	yes	not available	-2,15	-1,56
**9**	yes	NE	HP	no	no	yes	yes	frameshift	-1,49	-0,63
10	yes	yes	PPS, ASD, PDA, CoA	*yes*	no	yes	*yes*	missense	-0,93	0,79
**11**	yes	NE	PPS	*yes*	NE	yes	yes	frameshift	0,13	-1,83
**12**	yes	NE	PPS, ASD	*yes*	no	yes	yes	gene deletion	-1,64	-1,35
**13**	yes	NE	PPS	*yes*	*yes*	yes	yes	nonsense	-2,41	-1,31
**14**	yes	*no*	PPS	*yes*	no	yes	ND (after MLPA)		-2,47	-0,41
**15**	yes	*no*	PPS, VSD	*yes*	*yes*	yes	ND (after MLPA)		-1,7	-1,81
**16**	yes	yes	PPS	no	no	yes	ND (after MLPA)		-0,86	-1,55
**17**	yes	NE	PPS	*yes*	no	yes	under investigation		-2,27	-1,7
**18**	yes	*no*	PS ,VSD, ASD, PDA	*yes*	*yes*	yes	yes	missense	0,26	-1,3
**19**	yes	NE	TOF	*yes*	no	yes	yes	missense	-1,3	-2,69
20	yes	yes	PPS, ASD	*yes*	no	yes	*yes*	frameshift	1,48	1,34
**21**	yes	yes	PPS	*yes*	no	yes	yes	nonsense	0,06	-0,56
22	yes	yes	PPS	*yes*	*yes*	yes	*NE*		-2,65	0,46
**23**	yes	yes	PS	*yes*	*yes*	yes	yes	missense	-2,03	-0,59
**24**	yes	NE	PPS, ASD	*yes*	no	yes	yes	missense	-1,31	-0,64
**25**	yes	yes	PPS, VSD, ASD	*yes*	*yes*	yes	ND (after MLPA)		0,73	-1,48
**26**	yes	*no*	no	*yes*	*yes*	yes	yes	frameshift	-3,59	-1,16
**27**	yes	yes	PPS, PS	*yes*	*yes*	yes	yes	frameshift	-0,72	-0,55
**28**	yes	NE	VSD, ASD	*yes*	*yes*	yes	yes	gene deletion	-1,54	-0,31
**29**	yes	*no*	PPS	*yes*	*yes*	yes	yes	missense	-3,06	-1,12
**30**	yes	NE	PPS, PS, ASD, VSD	*yes*	*yes*	yes	yes	deletion of ex 20-23	0,42	-0,6
**31**	yes	NE	PPS	*yes*	*yes*	yes	yes	nonsense	-4,16	-0,96
32	*yes*	yes	PPS, ASD	no	*yes*	yes	*NE*		-3,2	2,02
33	*yes*	*no*	PPS	*yes*	*yes*	yes	yes	splice site	-1,88	-0,88

ASD - atrial septal defect, BMI - body mass index, CoA - aortic coarctation, HP - hypoplasia of the pulmonary arteries, MLPA - Multiplex Ligation-dependent Probe Amplification, ND - not detected, NE - not examined, PDA - peristant ductus aorticus, PPS - peripheral pulmonary stenosis, PS - pulmonary stenosis, VSD - ventricular septal defect, TOF - tetralogy of Fallot
